# Adipose tissue insulin resistance in young Japanese women is associated with metabolic abnormalities and dehydroepiandrosterone-sulfate

**DOI:** 10.3389/fendo.2024.1390778

**Published:** 2024-09-23

**Authors:** Motonori Sato, Yoshifumi Tamura, Hideyoshi Kaga, Nozomu Yamasaki, Satoshi Kadowaki, Daisuke Sugimoto, Takashi Nakagata, Yuki Someya, Yuya Nishida, Ryuzo Kawamori, Hirotaka Watada

**Affiliations:** ^1^ Department of Metabolism and Endocrinology, Juntendo University Graduate School of Medicine, Tokyo, Japan; ^2^ Sportology Center, Juntendo University Graduate School of Medicine, Tokyo, Japan; ^3^ Center for Therapeutic Innovations in Diabetes, Juntendo University Graduate School of Medicine, Tokyo, Japan; ^4^ Center for Identification of Diabetic Therapeutic Targets, Juntendo University Graduate School of Medicine, Tokyo, Japan

**Keywords:** underweight young women, adipose tissue insulin resistance, impaired glucose tolerance, metabolic abnormality, dehydroepiandrosterone-sulfate

## Abstract

**Objective:**

The proportion of young Japanese women who are underweight is exceptionally high. We previously showed that the prevalence of impaired glucose tolerance (IGT) was high in underweight young Japanese women, and that IGT was characterized by high free fatty acid levels and adipose tissue insulin resistance (ATIR). As the next step, this study aimed to explore factors associated with elevated ATIR in this population.

**Participants:**

Ninety-eight young, healthy, underweight women participated in this study.

**Design:**

To investigate the relationship between ATIR and metabolic parameters, participants were divided into three groups (Low, Medium, and High) according to ATIR level. Body composition examination, oral glucose tolerance testing, and blood biochemical analysis were performed; Adipo-IR and the Matsuda index were used as indices of ATIR and systemic insulin sensitivity, respectively.

**Results:**

Participants in the High ATIR group had the highest prevalence of IGT (25%), and significantly higher body fat percentage, whole-body insulin resistance, and levels of insulin-like growth factor-1 and dehydroepiandrosterone sulfate (DHEA-S) than the other two groups. They were also significantly younger and had higher systolic blood pressure than the Low ATIR group. Multiple regression analysis showed that DHEA-S, which is known to enhance lipolysis in adipose tissue, was an independent correlate of ATIR.

**Conclusions:**

Underweight Japanese women with high ATIR had impaired metabolism, a higher prevalence of IGT, higher systemic insulin resistance, and higher systolic blood pressure. DHEA-S was a determinant of high ATIR levels.

## Introduction

Insulin enhances the storage of triglycerides in adipose tissue, at least in part by suppressing the hydrolysis of triglycerides and the release of free fatty acids (FFAs) and glycerol (1). In obese individuals, the suppressive effect of insulin on lipolysis is insufficient, leading to elevated circulating levels of FFAs, a condition referred to as adipose tissue insulin resistance (ATIR) (2, 3). Excess FFAs, in turn, have been linked to insulin resistance in skeletal muscle and liver, in part through the accumulation of ectopic fat (4), supporting the lipotoxicity hypothesis. This adverse metabolic feature is observed in various obesity-associated diseases, including non-alcoholic fatty liver disease, metabolic syndrome, impaired glucose tolerance (IGT), and type 2 diabetes (5–8).

In the context of rising global obesity rates, it is often overlooked that underweight people in developed countries are not necessarily metabolically healthy. In Japan, for instance, the proportion of underweight (body mass index [BMI] ≤18.5 kg/m^2^) women in their 20s increased from 12.7% in 1982 to 19.8% in 2018, based on a National Nutrition Survey report. One of the main reasons for this is believed to be that young women want to lose weight (9, 10). This trend seems to contribute to a lower occurrence of abnormal glucose metabolism. However, a previous study showed that underweight women aged 40-79 years had almost twice the risk of developing type 2 diabetes than women of normal weight (11). Based on this report, we previously investigated glucose tolerance and metabolic parameters in underweight young Japanese women, because the ratio of underweight women in this generation is markedly higher than among women aged 40-79 years. Our data showed that underweight young women had a seven-fold higher prevalence of IGT than normal weight women (12). Furthermore, despite their low body mass index (BMI) and young age, these women had elevated FFA levels and high ATIR, in addition to impaired insulin secretion. However, the causal effects and specific characteristics of high ATIR in underweight young women remain largely unexplored (9).

Against this background, this study aimed to unveil the unique metabolic characteristics of underweight young women with high ATIR and explore the factors contributing to their heightened ATIR.

## Research design and methods

### Study subjects

We studied 98 young, healthy, underweight women with BMIs ranging from ≥16.0 to <18.5 kg/m^2^. Women were identified through two outsourcing companies (Souiken, Tokyo, Japan, and 3H medi solution, Tokyo, Japan). We excluded those with diabetes, hypertension, dyslipidemia, hyperthyroidism, surgical menopause, multipara, or any chronic disease. We also excluded those taking medicines or supplements that might affect metabolism, and those with suspected anorexia nervosa based on the Eating Attitude Test (EAT-26, Japanese version) (13). This study was approved by the ethics committee of Juntendo University (No. 2015045) and performed in accordance with the principles outlined in the Declaration of Helsinki. Written informed consent was obtained from all the subjects.

### Study design

This study was a sub-analysis of our previous study, whose design was already reported in detail (12). Briefly, all measurements were performed at the Juntendo Sportology Center (Tokyo, Japan) from November 2018 to December 2019. After fasting overnight, 98 participants underwent examinations in the morning on days 3-7 of their menstrual cycle to stabilize female hormone levels, which may affect glucose metabolism. Body composition was measured using the bioimpedance method (InBody; BIOSPACE) and dual-energy X-ray absorptiometry (DXA) (Hologic Discovery-A; Hologic, Inc., Bedford, MA). Blood samples were collected with patients in the supine position after at least a 15-minute rest, and then an 75-g oral glucose tolerance test (OGTT) was performed. We also administered the Brief-Type Self-Administered Diet History Questionnaire to assess energy intake (14), and the International Physical Activity Questionnaire short form to evaluate physical activity (15). Hand grip strength was measured using a hand grip dynamometer (Takei Digital Grip Strength Dynamometer; Takei Scientific Instruments Co., Ltd, Tokyo, Japan), and peak oxygen uptake was estimated with incremental exercise testing using a cycle ergometer (AEROBIKE 75XL; COMBI, Tokyo, Japan). After that, all subjects wore activity meters (AM-161; TANITA Co., Tokyo, Japan) for 7 days to examine physical activity in everyday life. We also asked about each subject’s previous maximum weight and changes in weight over the past year.

### Whole-body insulin resistance and ATIR

We evaluated whole-body insulin resistance using the Matsuda index (10,000/square root of [fasting glucose x fasting insulin] x [mean glucose x mean insulin during OGTT]) (16) and the homeostasis model assessment of insulin resistance (HOMA-IR; [fasting glucose x fasting insulin)/405]), and assessed ATIR using the fasting adipose tissue insulin resistance index (Adipo-IR) (fasting insulin x fasting FFA) (5).

### Biochemistry analysis

The level of every hormone examined was determined by SRL Laboratory (Tokyo, Japan) using a chemiluminescent enzyme immunoassay (insulin, dehydroepiandrosterone-sulfate [DHEA-S]), immunoradiometric assay (insulin-like growth factor 1 [IGF-1]), Clinical Laboratory Improvement Amendments assay (luteinizing hormone, follicle-stimulating hormone), and electrochemiluminescence immunoassay (estradiol).

### Statistical analysis

To investigate the characteristics of participants with high ATIR, we divided underweight participants into three groups (Low ATIR, Middle ATIR, and High ATIR) based on their levels of Adipo-IR. We used IBM SPSS Statistics for Windows, version 25.0. (IBM Corp., Armonk, NY, USA) for the analyses. Data are presented as means ± SD. Data were analyzed using the Kruskal-Wallis test for non-parametric comparison across multiple groups, followed by a *post-hoc* Dunn-Bonferroni test for pairwise comparisons. The correlations between parameters were assessed using the Spearman correlation coefficient. A multiple regression analysis was conducted to identify potential factors contributing to ATIR. The independent variables included in the analysis were %body fat, DHEA-S, and IGF-1 in Model 1, while Model 2 incorporated these variables plus age. This selection was guided by the outcomes of single-correlation analyses and previous research findings (17–19). All statistical tests were two-sided, with a significance level of 5%.

## Results

**Figure 1:**
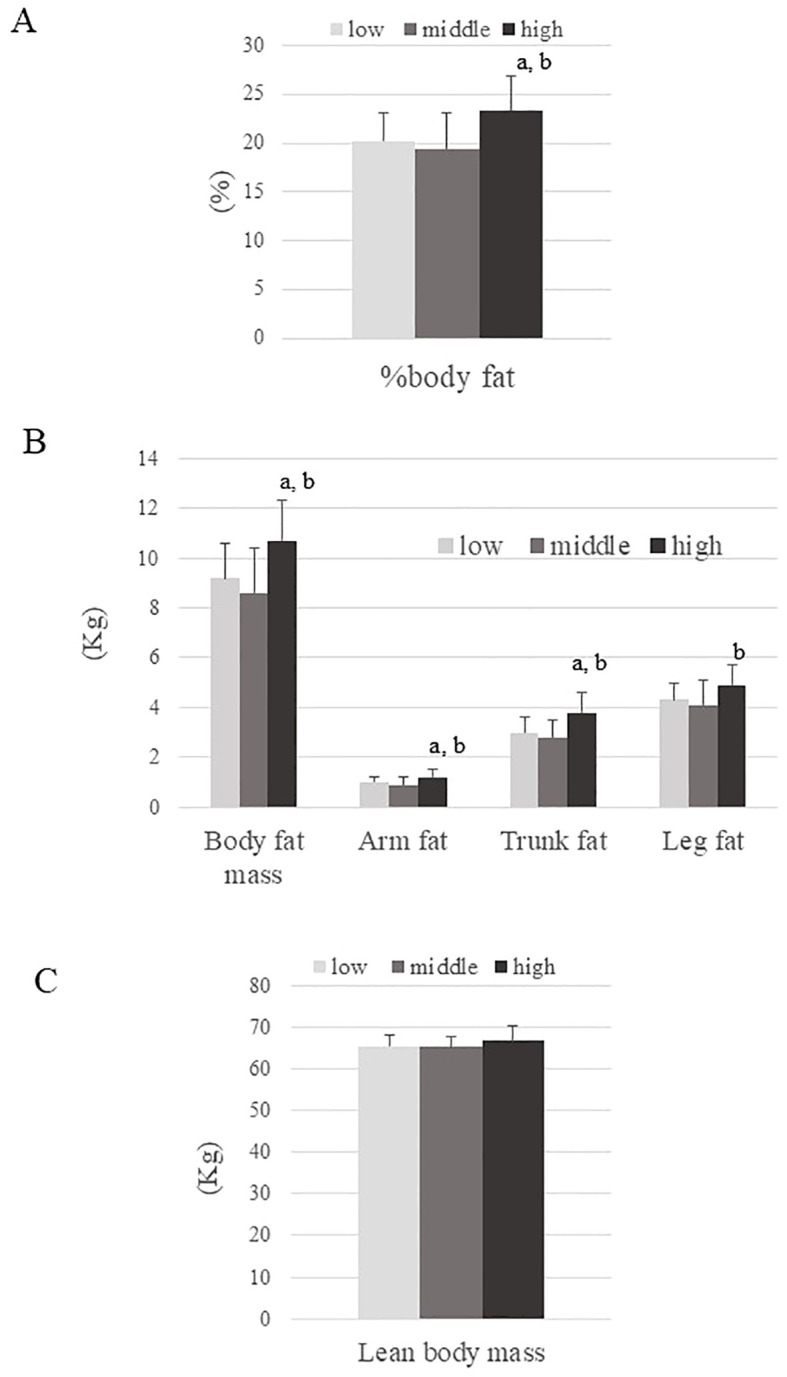
Body composition parameters across three ATIR groups (Low, Middle, and High). **(A)** Percentage of body fat, **(B)** Body fat mass, arm fat, trunk fat, and leg fat, **(C)** Lean body mass. P values are based on the Kruskal-Wallis test. a; P<0.05: for significant difference compared to the Low ATIR group. b; P<0.05: for significant difference compared to the Middle ATIR group.

The mean age and BMI of participants were 23.6 ± 3.0 years and 17.4 ± 0.7 kg/m^2^, respectively. **Table 1** summarizes the characteristics of the three groups defined by Adipo-IR level. Age was younger and systemic blood pressure was higher in the High ATIR group than in the Low ATIR group. While BMI and lean body mass were comparable between groups, the High ATIR group had the highest %body fat, body fat mass, arm fat, and trunk fat (**Figure 1**). In addition, the IGF-1 and DHEA-S levels were highest in the High ATIR group.

**Table 1:** Clinical characteristics of the Low ATIR, Middle ATIR, and High ATIR groups.

	Low ATIR (n=33)	Middle ATIR (n=33)	High ATIR (n=32)	*P*
Adipo-IR	1360 ± 408	2743 ± 525	8049 ± 7515	**-**
Age (y)	25.2 ± 3.0	23.5 ± 2.7	22.1 ± 2.8 ^a^	**<0.001**
Family history of diabetes (% (n))	24 (8)	24 (8)	28 (9)	0.998
Height (cm)	160.3 ± 5.1	158.5 ± 4.2	160.2 ± 6.1	0.346
Body weight (kg)	44.6 ± 2.8	43.5 ± 2.6	44.9 ± 3.8	0.210
Body mass index (kg/m^2^)	17.3 ± 0.6	17.3 ± 0.7	17.5 ± 0.7	0.433
%body fat (%)	20.2 ± 2.9	19.4 ± 3.7	23.4 ± 3.4 ^ab^	**<0.001**
Body fat mass (kg)	9.2 ± 1.4	8.6 ± 1.8	10.7 ± 1.6 ^ab^	**<0.001**
-Arm fat (kg)	1.0 ± 0.2	0.9 ± 0.3	1.2 ± 0.3^ab^	**<0.001**
-Trunk fat (kg)	3.0 ± 0.6	2.8 ± 0.7	3.8 ± 0.8^ab^	**<0.001**
-Leg fat (kg)	4.3 ± 0.7	4.1 ± 1.0	4.9 ± 0.8^b^	**0.003**
Lean body mass (kg)	34.4 ± 2.8	33.8 ± 2.5	33.3 ± 3.5	0.286
Waist (cm)	65.5 ± 3.9	65.3 ± 3.4	66.8 ± 4.4	0.477
Systolic blood pressure (mmHg)	103.2 ± 8.0	104.2 ± 8.6	108.5 ± 8.8 ^a^	**0.034**
Diastolic blood pressure (mmHg)	65.6 ± 8.7	64.6 ± 8.8	67.1 ± 7.1	0.398
VO_2_ peak (ml/min·kg)	34.3 ± 5.5	33.6 ± 4.1	32.3 ± 5.2	0.160
Total METs/week (mets·min/week)	37.2 ± 41.9	32.2 ± 51.3	28.8 ± 41.4	0.522
Total energy intake (kcal)	1470 ± 569	1223 ± 428	1306 ± 487	0.121
Protein intake (g)	52.1 ± 21.4	46.9 ± 22.4	45.4 ± 18.3	0.302
Fat intake (g)	45.7 ± 17.0	39.7 ± 17.9	42.0 ± 16.5	0.206
Carbohydrate intake (g)	201 ± 86	156 ± 54^a^	175 ± 71	**0.047**
Fasting glucose (mg/dl)	82.0 33± 5.9	84.7 ± 6.7	85.9 ± 8.4	0.060
Fasting insulin (μU/mL)	3.1 ± 1.2	4.3 ± 1.6	8.0 ± 6.0^ab^	**<0.001**
Fasting FFAs (µEq/L)	486.6 ± 194.0	694.5 ± 188.9^a^	1003.1 ± 343.5^ab^	**<0.001**
Glucose at 120 min during OGTT (mg/dl)	94.7 ± 19.7	100.4 ± 22.2	122.3 ± 34.6^ab^	**0.002**
Impaired glucose tolerance (% (n))	3 (1)	12 (4)	25 (8)	**0.014**
AUC glucose during OGTT (mg·min/dL·10^3^)	13.1 ± 2.5	14.1 ± 2.8	15.6 ± 3.5^a^	**0.008**
AUC insulin (µU·min/mL·10^3^)	4.3 ± 1.8	6.3 ± 3.2^a^	7.9 ± 3.1^ab^	**<0.001**
AUC FFA during OGTT (µEq·min/L·10^3^)	21.2 ± 6.4	23.6 ± 6.4	36.2 ± 16.1^ab^	**<0.001**
HbA1c (%)	5.2 ± 0.3	5.1 ± 0.2	5.2 ± 0.3	0.450
Insulinogenic index	1.04 ± 0.94	1.25 ± 0.97	2.00 ± 3.09	0.181
Disposition index	11.3 ± 11.2	9.5 ± 8.6	10.1 ± 18.0 ^a^	**0.014**
Matsuda index	11.7 ± 4.3	7.9 ± 2.6 ^a^	4.9 ± 1.7 ^ab^	**<0.001**
HOMA-β	68.3 ± 48.4	75.2 ± 28.4	169.1 ± 282.4 ^a^	**<0.001**
HOMA-IR	0.63 ± 0.25	0.91 ± 0.38 ^a^	1.77 ± 1.75 ^ab^	**<0.001**
High-density lipoprotein cholesterol (mg/dl)	68.8 ± 10.3	71.4 ± 16.6	66.7 ± 10.6	0.688
Low-density lipoprotein cholesterol (mg/dl)	98.1 ± 28.6	97.8 ± 25.8	106.5 ± 33.0	0.379
Triglyceride (mg/dl)	53.3 ± 26.4	46.0 ± 20.6	56.2 ± 20.9^b^	**0.021**
Adiponectin (µg/ml)	12.4 ± 5.2	13.3 ± 4.7	11.1 ± 4.9	0.143
Insulin-like growth factor 1 (ng/mL)	148.2 ± 43.1	146.8 ± 44.1	191.2 ± 53.4^ab^	**<0.001**
Luteinizing hormone (mIU/mL)	4.7 ± 2.5	4.6 ± 2.3	4.4 ± 2.0	0.994
Follicle-stimulating hormone (mIU/mL)	5.7 ± 1.4	6.1 ± 1.8	5.5 ± 1.5	0.617
Estradiol (pg/mL)	47.1 ± 26.1	70.5 ± 42.9^a^	45.3 ± 24.9^b^	**0.005**
Progesterone (ng/mL)	0.2 ± 0.2	0.2 ± 0.1	0.3 ± 0.2	0.257
Testosterone (ng/dL)	0.3 ± 0.1	0.3 ± 0.1	0.3 ± 0.1	0.705
Prolactin (μg/L)	19.0 ± 8.0	17.7 ± 7.3	23.3 ± 22.0	0.602
Adrenocorticotropic hormone (pg/mL)	21.1 ± 12.2	20.6 ± 10.6	25.6 ± 33.3	0.816
Cortisol (μg/dL)	12.9 ± 6.3	13.8 ± 5.9	14.4 ± 6.5	0.457
Dehydroepiandrosterone sulfate (μg/dL)	203.4 ± 63.5	204.7 ± 62.5	268.8 ± 104.7^ab^	**0.013**

Data are means ± SD. P values are based on the Kruskal-Wallis test. Bold text represents statistical significance (P<0.05). ^a^P<0.05: for significant difference compared to the Low ATIR group. ^b^P<0.05: for significant difference compared to the middle ATIR group.

ATIR, adipose tissue insulin resistance; Adipo-IR, adipose insulin resistance index; VO_2_peak, peak oxygen consumption; METs, metabolic equivalents; OGTT, oral glucose tolerance test; AUC, area under the curve; FFA, free fatty acid; HOMA-β, homeostasis model assessment of β-cell function; HOMA-IR, homeostasis model assessment of insulin resistance.

**Figure 2:**
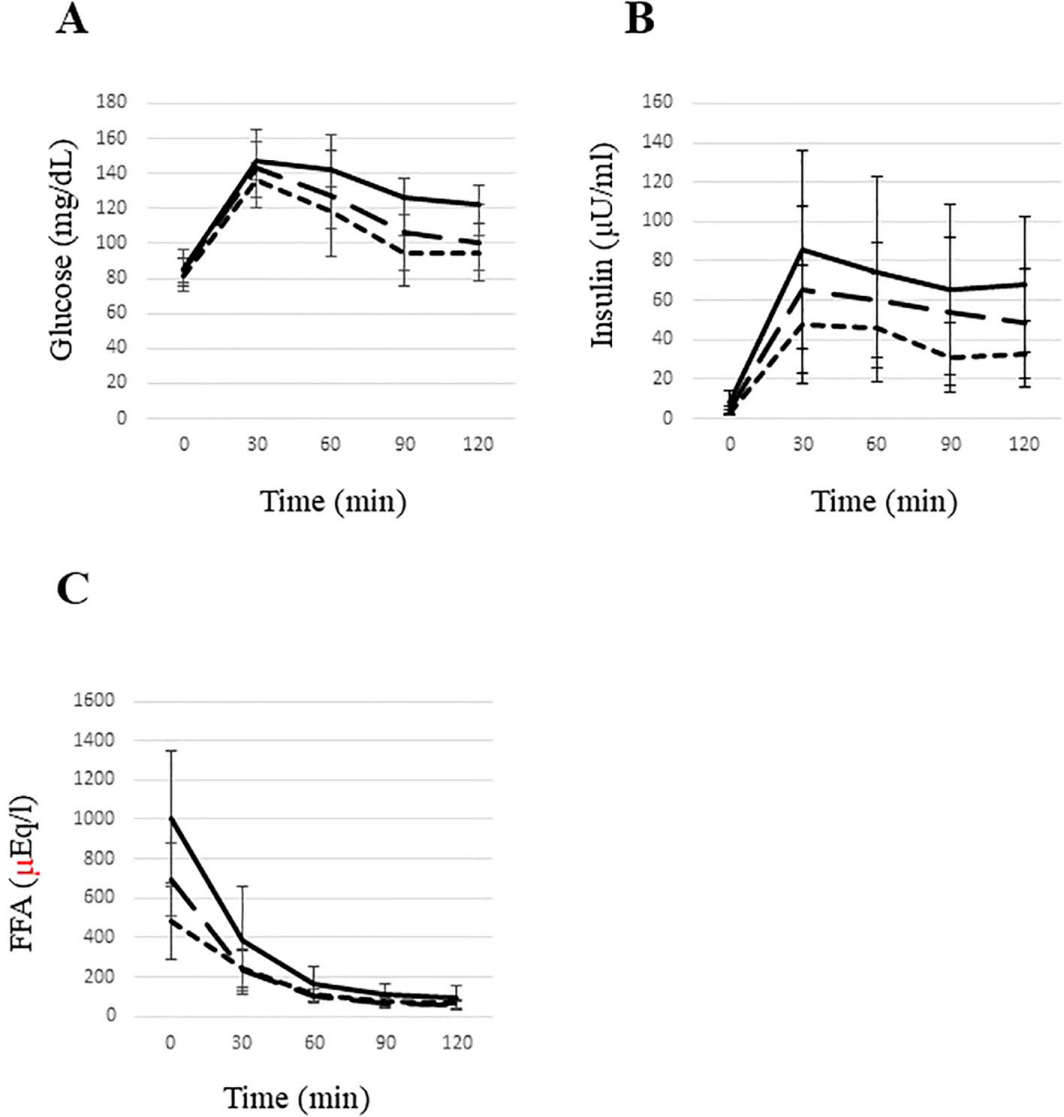
Glucose **(A)**, insulin **(B)**, and free fatty acid **(C)** levels during an oral glucose tolerance test in young underweight women. The area under the curve (AUC) for glucose **(A)** was significantly higher in the high ATIR group compared to the low ATIR group. The AUC for insulin **(B)** was significantly elevated in the high ATIR group compared to both the middle and low ATIR groups, with the middle ATIR group also showing a significantly higher AUC for insulin compared to the low ATIR group. Additionally, the AUC for FFAs **(C)** was significantly higher in the high ATIR group compared to both the low and middle ATIR groups. Statistical significance was determined using the Kruskal-Wallis test, with P<0.05 indicating significance. High ATIR, solid line; Medium ATIR, dashed line; Low ATIR, dotted line. FFA, free fatty acid.

While fasting glucose levels were comparable between groups, insulin and FFA levels were highest in the High ATIR group and were more than two-fold higher than in the Low ATIR group. The High ATIR group also had the highest values for several parameters: glucose level at 120 min, IGT prevalence, areas under the curve for insulin and FFA during OGTT, and indices of insulin resistance (Matsuda index and HOMA-IR). All of these parameters increased in order from the low ATIR group, to the Medium ATIR group, and then to the high ATIR group (**Table 1**, **Figure 2**).

**Table 2** shows the results of the single-correlation analysis of Adipo-IR. We investigated the correlation between Adipo-IR and the parameters that could be linearly related to changes in ATIR, as shown in **Table 1**. Adipo-IR was strongly correlated with both indices of insulin sensitivity (Matsuda index; r=-0.759, p<0.001, HOMA-IR; r=0.779, p<0.001). Adipo-IR was negatively correlated with age, and positively correlated with other parameters, specifically systolic blood pressure, body fat, HOMA-β, glucose level at 120 min during OGTT, DHEA-S level, and IGF-1 level.

**Table 2:** Spearman’s single-correlation analysis of Adipo-IR.

	r	*p*
Age	-0.401	**<0.001**
Systolic blood pressure	0.219	**0.030**
%body fat	0.312	**0.002**
Body fat mass	0.322	**0.001**
-Arm fat	0.314	**0.002**
-Trunk fat	0.310	**0.002**
-Leg fat	0.194	0.055
Fasting insulin	0.788	**<0.001**
Fasting glucose	0.254	**0.012**
Glucose level at 120 min during OGTT	0.359	**<0.001**
Fasting FFAs	0.718	**<0.001**
Dehydroepiandrosterone sulfate	0.364	**<0.001**
Insulin-like growth factor 1	0.351	**<0.001**
Matsuda index	-0.759	**<0.001**
HOMA-IR	0.779	**<0.001**
HOMA-β	0.645	**<0.001**

Bold text represents statistical significance (P<0.05).

OGTT, oral glucose tolerance test; FFA, free fatty acid; HOMA-β, homeostasis model assessment of β-cell function; HOMA-IR, homeostasis model assessment of insulin resistance.

Multiple regression analysis revealed that in Model 1, both the DHEA-S and IGF-1 levels were significantly and independently associated with Adipo-IR, but the IGF-1 level became non-significant after adjusting for age (Model 2) (**Table 3**).

**Table 3:** Multiple linear regression analysis of the relationship between Adipo-IR and each parameter.

	Adipo-IR	
	β	*p*
Model 1		
%fat	0.106	0.256
Dehydroepiandrosterone sulfate	0.345	**<0.001**
Insulin-like growth factor 1	0.239	**0.011**
Model 2		
Age	-0.131	0.212
%fat	0.098	0.295
Dehydroepiandrosterone sulfate	0.336	**<0.001**
Insulin-like growth factor 1	0.179	0.089

Bold text represents statistical significance (P<0.05).

## Discussion

In our previous study (12), we found that underweight young Japanese women with IGT had higher ATIR. However, the reason for this and the characteristics of underweight young women with high ATIR have been unclear. In the present study, we explored the features of underweight young women with high ATIR and found that the IGT prevalence and levels of glucose, insulin, C-peptide, and FFA during OGTT increased with higher ATIR (**Figure 2**, **Table 1**). We also found that subjects in the High ATIR group were younger and had higher body fat than those in the other two groups. Systolic blood pressure and the IGF-1 and DHEA-S levels were also highest in the High ATIR group. A significant correlation was observed between Adipo-IR and the above parameters. A multiple regression analysis revealed that the DHEA-S level was significantly and independently associated with Adipo-IR. While the DHEA-S levels were within the normal range across all groups, the significant correlation between ATIR and DHEA-S suggests that even normal physiological variations in DHEA-S can have a considerable impact on ATIR and metabolic health.

While normal ranges for Adipo-IR and Matsuda index in young Japanese women have not been definitively established, our previous study (12) provides some relevant data. In a preliminary calculation, we found that the mean Adipo-IR value in young Japanese women with normal weight was 3642 ± 2414, and the Matsuda index was 7.2 ± 2.8 (12). The values observed in the low and high ATIR groups in our current study were close to ±1SD of these mean values, indicating that the insulin resistance observed is within a borderline range rather than being extremely abnormal.

Our data clearly suggest that underweight women with high ATIR are moderately metabolically impaired. As mentioned in our previous study (12), IGT in underweight young women was associated with high ATIR and whole-body insulin resistance. Here, we showed that subjects with high ATIR had impaired glucose metabolism, whole-body insulin resistance, and slightly elevated blood pressure. Although the phenotype of high ATIR in young women seems to be less severe than that in obese subjects (20), moderate metabolic abnormalities in underweight young women could be caused by high ATIR. It is hypothesized that obesity causes metabolic abnormalities such as hypertension, glucose metabolic disorders, and dyslipidemia due to uncontrolled FFA release from adipose tissue and decreased insulin sensitivity. ATIR might cause moderate metabolic disorders even in underweight women (4–8). In the current study, the mean Adipo-IR in underweight women was 4010 ± 5149, which is slightly higher, though not significantly (p = 0.307 for Mann–Whitney U test), than the value observed in normal weight women (3642 ± 2414) (12). Although the differences in Adipo-IR between groups were not statistically significant, the observed trend suggests that elevated Adipo-IR might be one of the factors contributing to the higher prevalence of IGT in underweight young women, indicating a possible link between moderate insulin resistance and impaired glucose tolerance.

Body fat was highest in the High ATIR group (23.4 ± 3.4%) (**Figure 1**), suggesting that elevated body fat levels are one of the causes of ATIR. The higher ATIR in obese individuals is thought to be at least partly due to fat stores exceeding fat storage capacity, but the same may be true for certain non-obese individuals, especially Asians. In fact, previous reports showed that East Asians have a lower fat storage capacity in their adipose tissue compared to other ethnicities (21, 22). We previously reported that even apparently healthy non-obese Japanese men with moderate ATIR had higher body fat than controls (22.1 ± 4.7% vs. 18.6 ± 4.6%, respectively) (23). However, the correlation between percent body fat and Adipo-IR was found to be relatively weak (r=0.312, p<0.01), and it was not statistically significant in multiple regression analysis. This suggests that other factors (e.g. genetic predispositions) may be responsible for causing ATIR in underweight young women. Our findings also emphasize the need for further studies with larger sample sizes to confirm these observations and to explore additional factors contributing to ATIR in this population.

The High ATIR group was younger and had higher IGF-1 levels than the other two groups. Given that the IGF-1 level correlates with the growth hormone level, which is known to stimulate lipolysis in white adipose tissue (17, 18, 24), our findings suggest that higher IGF-1 levels might contribute to increased ATIR by promoting lipolysis through the actions of growth hormone. This is further corroborated by the fact that IGF-1 levels typically decrease with age, which was confirmed in our study by the significant negative correlation between IGF-1 level and age (r=-0.49, p ≤ 0.001). However, after adjusting for age, the correlation between IGF-1 level and Adipo-IR became less pronounced. This might indicate that although the role of IGF-1 in influencing Adipo-IR should not be disregarded, its impact may not be as substantial as initially anticipated. This observation also raises the possibility that ATIR may represent a transient metabolic impairment in younger individuals, which could potentially improve with age. However, longitudinal studies are needed to determine whether this is indeed the case.

The High ATIR group exhibited the highest DHEA-S level, a finding that seems contradictory to some previous studies suggesting that DHEA administration reduces adiposity and enhances insulin sensitivity in older people (25, 26). However, these studies predominantly focused on older individuals, who have naturally lower DHEA-S levels than young people, and thus the effects of DHEA-S in younger populations have been less explored. In line with this, an *in vitro* study demonstrated that DHEA-S promoted lipolysis in women’s subcutaneous adipose tissue (19). In addition, an *in vivo* and *in vitro* study demonstrated that DHEA promoted lipid mobilization in adipose tissue by increasing the expression and activity of adipose triglyceride lipase and hormone-sensitive lipase (27). Taken together, these data suggest that elevated DHEA-S levels can lead to increased FFA levels. Of note, high DHEA-S levels are observed in polycystic ovary syndrome, which is generally characterized by visceral fat accumulation and insulin resistance (28, 29). However, even in the High ATIR group, DHEA-S levels were mostly within the normal range. Another possible explanation for the high DHEA-S level in the High ATIR group is that an increase in FFAs might precede a rise in DHEA-S. A previous study reported that FFA elevation by lipid infusion enhanced the production of DHEA prior to the induction of insulin resistance (30). Thus, it is also possible that high DHEA-S levels may be due to increased levels of FFAs secondary to other causes.

Our study had several limitations. First, our findings indicate that young women who were underweight, a current social problem in Japan, and who had high ATIR exhibited some metabolic impairment; however, we cannot generalize these results to a genetically distinct population. Second, our study suggests that women with high ATIR tend to be younger, but the long-term implications of this condition on their metabolic health remain unknown. Furthermore, we did not investigate glucose tolerance in older age groups (30-50 years), which limits the scope of our findings. It is also important to consider that while energy intake and dietary composition were similar across groups, extreme dieting practices (e.g. intermittent fasting) were not specifically excluded, which could potentially influence ATIR. To fully elucidate the relationship between ATIR and age, it is crucial to conduct longitudinal studies.

In conclusion, underweight Japanese women with high ATIR were metabolically impaired, with a higher prevalence of IGT, higher whole-body insulin resistance, and higher systolic blood pressure. High ATIR was associated with younger age and higher levels of body fat, DHEA-S, and IGF-1, and DHEA-S might be a key factor affecting ATIR. However, the absence of longitudinal data and the lack of genetic predispositions represent limitations of this study. More detailed studies are necessary to better understand these relationships.

## Data Availability

The raw data supporting the conclusions of this article will be made available by the authors, without undue reservation.
